# Molecular Diagnosis of Cause of Anisakiasis in Humans, South Korea

**DOI:** 10.3201/eid2102.140798

**Published:** 2015-02

**Authors:** Hyemi Lim, Bong-Kwang Jung, Jaeeun Cho, Thanapon Yooyen, Eun-Hee Shin, Jong-Yil Chai

**Affiliations:** Seoul National University College of Medicine, Seoul, South Korea (H. Lim, B.-K. Jung, J. Cho, E.H. Shin, J.-Y. Chai);; Thaksin University, Phatthalung, Thailand (T. Yooyen)

**Keywords:** *Anisakis pegreffii*, *Anisakis simplex* sensu stricto, anisakiasis, human, fish, Korea

## Abstract

Anisakiasis in humans in South Korea has been considered to be caused exclusively by the larvae of *Anisakis simplex* sensu stricto and *Pseudoterranova decipiens.* Recently, however, DNA sequencing of larvae from 15 of 16 anisakiasis patients confirmed the cause to be *Anisakis pegreffii* infection*.* Molecular analysis should be performed for all extracted larvae.

Anisakiasis is a zoonotic nematode infection that causes acute and chronic gastrointestinal granulomatous disease in humans. For most patients, the causative agents are larvae of nematodes of the genera *Anisakis* and *Pseudoterranova*, and the source of infection is marine fish or squids harboring these larvae (*1*). Within 8–12 hours after infected fish are ingested, the larvae penetrate into the person’s stomach or intestinal wall, causing acute abdominal pain, indigestion, nausea, and vomiting; pathologic findings are edema, hyperemia, and bleeding in the surrounding mucosa (*1*,*2*). The diagnosis is usually based on morphologic identification of the larvae or on histopathologic identification of sectioned larvae (*1*). However, molecular techniques have recently been developed as effective tools not only for the diagnosis of individual cases but also for studies of taxonomy and evolution of anisakid nematodes (*3*,*4*).

Anisakiasis in humans was first reported in the Netherlands; since then, it has been reported extensively in Japan (≈2,000 cases annually), South Korea (≈200 cases annually), and some European countries (≈500 cases annually) where people eat raw or undercooked fish (*1*,*2*). In the United States, up to 50 human cases are reported each year (*1*). Most infections in humans have been caused by *Anisakis simplex* sensu stricto and *Pseudoterranova decipiens* nematodes (*1*); however, since 1999, a few human infections with *Anisakis pegreffii* larvae (a sibling species of *A. simplex* s.s.), originally recovered from a Mediterranean monk seal (*5*), have been reported in Italy (*6*–*9*) and Japan (*10*,*11*). The larvae of *A. pegreffii* are morphologically distinguished, with difficulty, from those of *A. simplex* s.s. (both are *Anisakis* type I); however, molecular techniques can easily distinguish the 2 types of larvae (*3*,*4*).

In South Korea, *Anisakis* type I larvae recovered from humans and fish have been assigned to *A. simplex* s.s., on the basis of morphologic appearance (*1*,*12*). We performed molecular analyses of 26 *Anisakis* type I larvae recovered from 16 humans in South Korea by using DNA sequencing of the nuclear internal transcribed spacer (ITS) genes.

## The Study

A total of 30 *Anisakis* type I larvae were removed from the stomach of 16 patients referred to the Department of Parasitology and Tropical Medicine, Seoul National University College of Medicine, Seoul, South Korea, from 2000 through 2013 (Table). Among them, 26 larvae were analyzed by DNA sequencing. All patients experienced acute gastric or abdominal discomfort, including epigastric pain and indigestion, and underwent gastroduodenoscopy. During the examinations, whitish nematode larvae were observed and extracted with biopsy forceps. Some larvae were preserved in 70% ethanol, and others were fixed in 10% formalin before being mounted on slides with glycerin jelly.

**Table:** Anisakiasis characteristics among 16 human patients, South Korea, 2000–2013*

Patient no.	Patient sex	Year of larvae recovery	No. larvae recovered	Clinical signs and symptoms	*Anisakis* larvae sequencing results (% identical sites)	
					*A. simplex* s.s.	*A. pegreffii*
1	M	2000	1	Abdominal pain, nausea	99.2	100
2	M	2000	1	Indigestion, vague gastric pain	99.2	100
3	M	2002	1	Abdominal pain, nausea, vomiting	99.2	100
4	F	2002	1	Abdominal pain and tenderness, anorexia	99.2	100
5	F	2003	1	Epigastric pain, nausea, vomiting	99.2	100
6	M	2003	1	Abdominal pain, indigestion	99.2	100
7	F	2003	1	Abdominal pain, nausea, diarrhea	99.2	100
8	F	2003	1	Abdominal pain, nausea, anorexia	99.2	100
9	F	2004	1	Epigastric pain, abdominal fullness	99.2	100
10	M	2005	1	Abdominal pain, nausea, vomiting	99.2	100
11	F	2005	1	Abdominal pain, indigestion, nausea, vomiting	99.2	100
12	M	2005	1	Abdominal pain, nausea, vomiting	99.2	100
13	M	2005	1	Epigastric pain, indigestion, nausea	99.2	100
14	M	2006	1	Abdominal discomfort, nausea	99.2	100
15	M	2012	1	Abdominal pain and tenderness	100	99.2
16	M	2013	15†	Abdominal pain and tenderness, indigestion, nausea, vomiting, anorexia	99.2	100

*All patients underwent gastroduodenoscopy, during which *Anisakis* larvae were removed with biopsy forceps. †Of these 15 larvae, 11 were analyzed by use of molecular techniques.

Total genomic DNA was extracted by using a DNeasy Blood and Tissue Kit (QIAGEN, Hilden, Germany); nested PCR and nucleotide sequencing were performed on the ITS region (ITS1, 5.8S rRNA subunit, and ITS2) according to procedures reported previously (*13*). The PCR product was amplified by using the Cosmo Labopass X2 PCR Premix kit (Cosmo Genetech, Seoul, South Korea), and automated DNA sequencing was performed by Solgent Co., Ltd. (Daejeon, South Korea). Nucleotide sequences obtained were aligned by using the Geneious program, version 6.0.3 (Geneious Co., Wellington, New Zealand).

Of the 26 *Anisakis* larvae from 15 human patients, 25 showed 100% identity in the sequences of ITS region (244 bp, high-confidence variable positions) with those of the *A. pegreffii* sequence available in GenBank (accession no. AB277823), whereas their identity with *A. simplex* s.s. (accession no. AB277822) was 99.2% (Table). The remaining sample showed 100% identity with the sequences of *A. simplex* s.s. (accession no. AB277822) and 99.2% identity with those of *A. pegreffii* (accession no. AB277823). On the basis of these results, *A. pegreffii* nematode infection was diagnosed for 15 of the 16 patients, and *A. simplex* s.s. infection was diagnosed for only 1 patient.

## Conclusions

Our results confirm the presence of *A. pegreffii* nematode infection in humans in South Korea, making this the third country (after Italy and Japan) in which this infection in humans has been identified. This high proportion of *A. pegreffii* nematode infections in humans is surprising and suggests that most cases of anisakiasis in humans in South Korea may be caused by *A. pegreffii* rather than *A. simplex* s.s. larvae. To confirm the source of infection, molecular analyses of *Anisakis* larvae extracted from human patients are required in South Korea.

Human infection with *A. pegreffii* nematodes was first documented in Italy by use of PCR-based restriction fragment length polymorphism (PCR-RFLP) analysis (*6*). The second case was reported from Japan (*10*), in which 1 of 100 anisakid larvae extracted from patients in Kyushu and Hokkaido was identified by PCR-RFLP analysis as *A. pegreffii*. Then, in 2009, *A. pegreffii* nematode infection was diagnosed for 2 women in Italy by PCR-RFLP and sequencing of the 28S gene (*7*). In 2011, *A. pegreffii* DNA was extracted from a paraffin-embedded granuloma from a man in Italy (*8*). Also in Italy, 8 more *A. pegreffii* nematode infections in humans were reported in 2013 (*9*). Thus, to date, including the 15 cases reported here, a total of 28 cases of *A. pegreffii* nematode infections in humans have been documented in the literature.

The markedly high proportion of *A. pegreffii* nematode infections identified among patients in South Korea (25/26 larvae from 15/16 patients) was not expected because in Japan (Kyushu and Hokkaido), which are geographically close to South Korea, *Anisakis* larvae from humans are mostly *A. simplex* s.s. (99/100 larvae from 84/85 patients); only 1 larva was identified as *A. pegreffii* (*10*). This remarkable discrepancy between South Korea and Japan remains to be further investigated. However, it is of note that the species of *Anisakis* larvae detected in fish varied according to the 2 large localities of Japan; from northern Japan to the Pacific sides and from the Sea of Japan to the eastern China Sea sides (*14*). The former locality, such as Hokkaido and eastern Japan, showed more *A. simplex* s.s. than *A. pegreffii* larvae, whereas the latter locality (southwestern Japan), including Kyushu and Fukuoka (close to South Korea), showed more *A. pegreffii* than *A. simplex* s.s larvae (*14*). This finding might partly explain the discrepancy between the *Anisakis* larvae species that infect humans in South Korea and Japan.

When the pathogenic potential of *A. simplex* s.s. larvae for human patients was compared with that of *A. pegreffii* (*11*), it was found that *A. simplex* s.s. larvae had greater potential than *A. pegreffii* larvae to survive acidic gastric juice and to penetrate the human stomach, small intestine, and colon. However, further studies are needed to elucidate this finding.

Another clinicopathologic concern associated with anisakiasis in humans is the potential for *A. simplex* s.s. and *A. pegreffii* larvae to elicit gastroallergic reactions. These reactions are characterized by urticaria on the arms and abdomen and by angioedema or anaphylaxis when the live parasite attempts to penetrate the gastric mucosa (*9*). We did not notice such allergic reactions in the patients reported here. However, because of an increasing tendency toward *Anisakis* nematode allergy among patients in South Korea (*15*), attention should be paid to this clinical feature.

Our study demonstrates the predominance of *A. pegreffii* over *A. simplex* s.s. nematode infection among humans with anisakiasis in South Korea. The study highlights the need to perform molecular analysis for each larva extracted from human patients in this country.

## References

[1] Sohn WM, Chai JY. Anisakiosis (anisakidosis). In: Palmer SR, Soulsby L, Torgerson PR, Brown WG, editors. Oxford textbook of zoonoses. 2nd ed. Oxford (UK): Oxford University Press; 2011. p. 774–86.

[2] Chai JY, Murrell KD, Lymbery AJ. Fish-borne parasitic zoonoses: status and issues. Int J Parasitol. 2005;35:1233–54. 10.1016/j.ijpara.2005.07.013 16143336

[3] Mattiucci S, Nascetti G. Advances and trends in the molecular systematics of anisakid nematodes, with implications for their evolutionary ecology and host-parasite co-evolutionary processes. Adv Parasitol. 2008;66:47–14. 10.1016/S0065-308X(08)00202-9 18486689

[4] Mattiucci S, Cipriani P, Webb SC, Paoletti M, Marcer F, Bellisario B, Genetic and morphological approaches distinguish the three sibling species of the *Anisakis simplex* species complex, with a species designation as *Anisakis berlandi* n. sp. for *A. simplex* sp. C (Nematoda: Anisakidae). J Parasitol. 2014;100:199–21. 10.1645/12-120.1 24224764

[5] Campana-Rouget PY, Biocca E. Une nouvelle espéce d’*Anisakis* chez un phoque Mediterranean Annales de Parasitologie. 1955;30:477–80. 13327690

[6] D’Amelio S, Mathiopoulos KD, Brandonisio O, Lucarelli G. Doron zo F, Paggi L. Diagnosis of a case of gastric anisakidosis by PCR-based restriction fragment length polymorphism analysis. Parassitologia. 1999;41:591–3 . 10870568

[7] Fumarola L, Monno R, Ierardi E, Rizzo G, Giannelli G, Lalle M, *Anisakis pegreffii* etiological agent of gastric infections in two Italian women. Foodborne Pathog Dis. 2009;6:1157–9. 10.1089/fpd.2009.0325 19642920

[8] Mattiucci S, Paoletti M, Borrini F, Palumbo M, Palmieri RM, Gomes V, First molecular identification of the zoonotic parasite *Anisakis pegreffii* (Nematoda: Anisakidae) in a paraffin-embedded granuloma taken from a case of human intestinal anisakiasis in Italy. BMC Infect Dis. 2011;11:1–6. 10.1186/1471-2334-11-82 21453522 PMC3080813

[9] Mattiucci S, Fazii P, Rosa AD, Paoletti M, Megna AS, Glielmo A, Anisakiasis and gastroallergic reactions associated with *Anisakis pegreffii* infection, Italy. Emerg Infect Dis. 2013;19:496–9 10.3201/eid1903.121017. 10.3201/eid1903.121017 23621984 PMC3647659

[10] Umehara A, Kawakami Y, Araki J, Uchida A. Molecular identification of the etiological agent of the human anisakiasis in Japan. Parasitol Int. 2007;56:211–5. 10.1016/j.parint.2007.02.005 17428725

[11] Arizono N, Yamada M, Tegoshi T, Yoshikawa M. *Anisakis simplex* sensu stricto and *Anisakis pegreffii*: biological characteristics and pathogenetic potential in human anisakiasis. Foodborne Pathog Dis. 2012;9:517–21. 10.1089/fpd.2011.1076 22545961

[12] Chai JY, Cho SR, Kook J, Lee SH. Infection status of the sea eel (*Astroconger myriaster*) purchased from the Noryangjin fish market with anisakid larvae [in Korean]. Korean J Parasitol. 1992;30:157–62. 10.3347/kjp.1992.30.3.157 1420027

[13] Suzuki J, Murata R, Hosaka M, Araki J. Risk factors for human *Anisakis* infection and association between the geographic origins of *Scomber japonicus* and anisakid nematodes. Int J Food Microbiol. 2010;137:88–93. 10.1016/j.ijfoodmicro.2009.10.001 19892425

[14] Quiazon KMA, Yoshinaga T, Ogawa K. Distribution of *Anisakis* species larvae from fishes of the Japanese waters. Parasitol Int. 2011;60:223–6. 10.1016/j.parint.2011.03.002 21397715

[15] Choi SJ, Lee JC, Kim MJ, Hur GY, Shin SY, Park HS. The clinical characteristics of *Anisakis* allergy in Korea. Korean J Intern Med. 2009;24:160–3. 10.3904/kjim.2009.24.2.160 19543498 PMC2698627

